# Chronic Adult-Onset Still’s Disease With Positive Antinuclear Antibodies: Navigating Diagnostic Dilemmas and Clinical Implications

**DOI:** 10.7759/cureus.56776

**Published:** 2024-03-23

**Authors:** Keshao Nagpure, Prasanth Raju, Amol H Dube, Ishan Verma, Sunita Kumbhalkar

**Affiliations:** 1 General Medicine, All India Institute of Medical Sciences Nagpur, Nagpur, IND

**Keywords:** adult onset stills disease with positive antinuclear antibody(ana), chronic adult onset still's disease, systemic lupus erythromatosus, antinuclear antibody (ana), adult onset still's disease (aosd)

## Abstract

Adult-onset Still's disease (AOSD) is a rare systemic autoinflammatory disorder characterized by fever, rash, and joint pain. Despite primarily affecting young adults, it can occur at any age, presenting diagnostic challenges due to its heterogeneous nature and lack of specific laboratory findings. The subset of AOSD with positive antinuclear antibody (ANA) adds complexity, potentially overlapping with other autoimmune conditions. We describe a case of a 30-year-old female with a two-year history of fever, weight loss, and joint pain, initially misdiagnosed as seronegative arthritis with hypothyroidism. Further evaluation revealed severe anemia, leucocytosis, and hepatosplenomegaly. Despite a strongly positive ANA, the absence of systemic lupus erythematosus (SLE) features led to a diagnosis of chronic AOSD. Treatment with steroids and disease-modifying antirheumatic drugs (DMARDs) resulted in clinical improvement, highlighting the importance of accurate disease classification for tailored management in ANA-positive AOSD. This case underscores the diagnostic challenges of AOSD and emphasizes the need for precise classification for optimal treatment strategies.

## Introduction

In 1897, George Frederic Still described juvenile idiopathic arthritis (JIA) in 22 children, followed by Bywaters, who later defined adult-onset Still's disease (AOSD) [1,2]. AOSD is a rare autoimmune condition of unknown etiology, clinically characterized by a maculopapular rash, fever, arthralgia, leukocytosis, and elevated acute phase reactants. Diagnosis involves excluding a broad spectrum of infectious, autoimmune, and neoplastic pathologies, with Yamaguchi's criteria serving as a guideline [3]. While no specific diagnostic laboratory test exists, serum ferritin levels can aid in monitoring and considering AOSD [4]. Treatment primarily consists of non-steroidal anti-inflammatory drugs (NSAIDs), corticosteroids, and rheumatological agents [5]. Negative antinuclear antibody (ANA) and Rheumatoid (RA) factor are included as part of the minor criteria for diagnosing AOSD; however, rare cases with positive ANA and no other features of systemic lupus erythematosus (SLE) or other autoimmune disorders have been reported, suggesting that a positive ANA or RA factor does not rule out the diagnosis of AOSD [6]. Here, we present a case of AOSD diagnosed based on the Yamaguchi criterion and positive ANA.

## Case presentation

**Figure 1:**
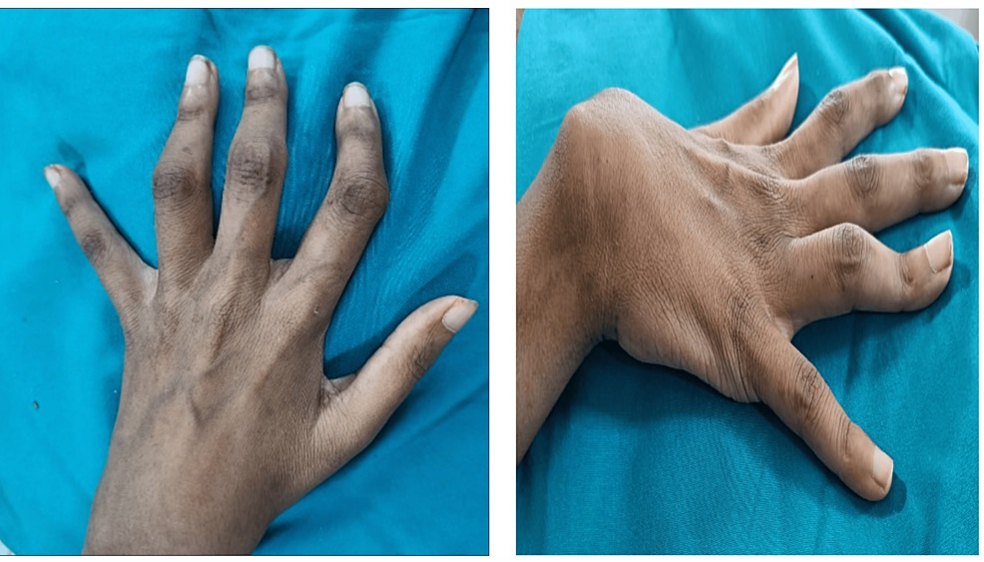
Patient's left and right hand images Left-hand side image: Boutonniere deformities in the left middle and index finger with radial deviation of the left index finger at the proximal interphalangeal joint with, Right-hand side image: Boutonniere deformities of the right ring finger with ulnar subluxation of the right wrist joint.

A 30-year-old female presented with a two-year history of high-grade fever (39.1°C) accompanied by chills, significant weight loss, and alopecia, as well as inflammatory joint pain for one year with associated hand deformity over eight months. She had a history of recurrent admissions and was evaluated as seronegative arthritis with hypothyroidism. Physical examination revealed signs of systemic involvement, including tachycardia, severe pallor, recently developed bilateral cervical lymphadenopathy, tender wrists, ankles, and small hand joints with bilateral phalangeal deformities (Figure 1).

Abdominal examination demonstrated grade 2 splenomegaly, and no hepatomegaly clinically. Laboratory findings are consolidated in Table 1. A complete hemogram was suggestive of severe microcytic anemia (hemoglobin-5.3 g/dl, mean corpuscular volume (MCV)-63.8 fl) with neutrophilic leucocytosis (total leukocyte count-26000 cells/mm^3^, neutrophil-88.8%) and normal platelet counts. Liver and kidney function tests were normal. Viral markers (HIV, Hepatitis B surface antigen (HBsAg), and Hepatitis C virus (HCV)) and tropical fever panel were negative. Cervical lymph node fine needle aspiration cytology (FNAC) and biopsy were suggestive of reactive lymphadenitis. Erythrocyte sedimentation rate (ESR), C-reactive protein (CRP), and lactate dehydrogenase (LDH) were raised (51 mm/hr, 186 mg/dl, and 899 U/L, respectively). The patient was negative for Rheumatoid factor and anti-cyclic citrullinated peptide (anti-CCP). Procalcitonin was raised (>16 ng/ml) and ferritin was very high (>1650 ng/dl). Further investigations for spondyloarthropathy and sacroiliac tenderness yielded borderline positive HLA-B27, but the MRI of the hip was unremarkable. Her thyroid stimulating hormone (TSH) was 97.43 mIU/ml, and anti-thyroid peroxidase (TPO) was > 1300 U/ml. The patient’s ANA was 1:320 positive with the speckled pattern but the ANA blot was negative. There was no specific antibody positive for SLE or other systemic features suggestive of SLE and Complement levels were normal.

**Table 1:** Laboratory findings of the patient TPO: Thyroid peroxidase.

Biochemical Parameter	On Admission	Post-treatment	Reference Range
Hemoglobin	5.3 g/dl	10 g/dl	12-15 g/dl
Total leukocyte count	26490 cells/mm^3^	9490 cells/mm^3^	4000-10,000 cells/mm^3^
Platelets	2.45 lakh/mm^3^	2.58 lakh /mm^3^	1.5-4.5 lakh/mm^3^
Erythrocyte sedimentation rate (ESR)	51 mm /hr	41 mm/hr	0-15 mmHg/hr
C-Reactive protein	186 mg/L	10.5 mg/L	<5 mg/L
Serum ferritin	>1600 ng/dl	350 ng/dl	13-150 ng/dl
Procalcitonin	> 16 ng/ml	0.21 ng/ml	<0.5 ng/ml
Spot urine albumin creatinine	150 mg/g	14 mg/g	< 30 mg/g
Lactate dehydrogenase	899 U/L	-	135-214 U/L
Serum creatinine	0.7 mg/dl	-	0.6-1.3 mg/dl
Aspartate aminotransferase (AST)	36.4 U/L	-	0-45 U/L
Alanine aminotransferase (ALT)	7.1 U/L	-	0-45 U/L
Thyroid stimulating hormone (TSH)	97.43 mIU/ml	-	0.27-4.20 mIU/ml
Anti-TPO (U/ml)	>1300 U/ml	-	Up to 60 Unit/ml
Complement C3 (C3)	0.91 g/L	-	0.80-1.70 g/L
Complement C4 (C4)	0.18 g/L	-	0.12-0.36 g/L
Antinuclear antibody (indirect-immunofluorescence)	Positive ++ (1:320, Speckled pattern)	-	Negative (No Immunofluorescence)
Antinuclear antibody (ANA) Blot	Negative	-	Negative (No antibody detected)
Rheumatoid factor (qualitative)	Negative	-	Negative
Direct Coomb’s Test & Indirect Coomb’s Test	Negative	-	Negative
Brucella serology	Negative	-	Negative
Blood and urine cultures	No growth		No-growth

**Figure 2:**
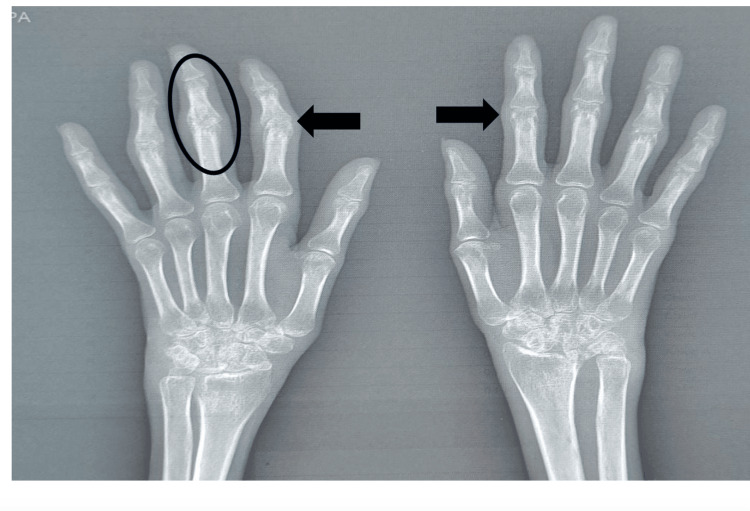
X-ray images of both hands The image shows reduced joint space, subchondral osteopenia, articular marginal erosions in proximal interphalangeal joints bilaterally, and ankylosis of the third PIP joint of the right hand.

Imaging studies revealed hepatosplenomegaly with multiple subcentimetric (<1 cm at the short axis, non-necrotic) mesenteric lymph nodes and bilateral minimal pleural effusion. X-rays of the hands showed evidence of joint damage, including reduced joint space, subchondral osteopenia, and articular marginal erosions in the proximal interphalangeal joints, with ankylosis of the third proximal interphalangeal joint of the right hand (Figure 2).

As per Yamaguchi criteria, the patient had five positive criteria, of which three were major criteria (fever > 39°C for > 1 week, arthritis for > two weeks, and persistent leucocytosis of 26000 cells/ mm^3^ with polymorphonuclear cells of 88%) and two minor criteria (recent development of significant lymphadenopathy and hepatosplenomegaly), and therefore was diagnosed with AOSD. The clinical pattern of AOSD was chronic, as the duration of the disease was >1 year. The patient was treated with prednisone 60 mg/day, naproxen 500 mg/day, and methotrexate 15 mg/week with folic acid 5 mg twice a week. At the follow-up, corticosteroids were gradually decreased as symptoms, such as fever, and arthralgia, showed improvement, and was continued on tapering doses of steroids along with Methotrexate and folic acid supplement.

## Discussion

Adult-onset Still's Disease (AOSD) is a rare autoimmune disorder that affects multiple systems in the body. Despite its rarity, diagnosing AOSD can be challenging due to its diverse clinical presentation and the absence of specific diagnostic tests. Globally, the illness primarily affects young adults (the median age at diagnosis is approximately 36 years) [7,8]; but cases have been reported as late as 83 years [9,10]. While the exact cause of AOSD remains unknown, researchers have identified elevated levels of certain inflammatory markers, including tumor necrosis factor-alpha (TNF-α), interleukin-6 (IL-6), and interleukin-18 (IL-18), in individuals with the condition [5]. These elevated markers are believed to contribute to the systemic inflammation seen in AOSD.

Typically, individuals with AOSD experience symptoms such as high-grade fever, rash, joint pain (arthralgia), and sore throat, as seen in this case, though rash and sore throat were absent. The characteristic rash associated with AOSD is described as a macular or maculopapular evanescent salmon-pink skin rash that appears and disappears during fever spikes. It is predominantly found on the proximal limbs and trunk [11,12]. Apart from this, there can be lymphadenopathy, and splenomegaly, which was also seen in our case.

The diagnosis of AOSD is based on Yamaguchi's criteria (Table 2), which necessitates the presence of five or more criteria, including at least two major criteria, achieving a sensitivity of 96.2% and specificity of 92.1%. Excluding other disorders is also a key component of the diagnostic process [3].

**Table 2:** Yamaguchi criteria for diagnosis of adult-onset Still’s disease Five or more criteria are required, of whom two or more must be major criteria. Reference no. [3]

Major criteria	Minor criteria
Fever > 39^◦^C, lasting one week or longer	Sore throat
Arthralgia or arthritis, lasting two weeks or longer	Recent development of significant lymphadenopathy
Typical rash	Hepatomegaly or splenomegaly
Leucocytosis > 10000 cells/mm^3^ with > 80% polymorphonuclear cells	Abnormal liver function tests
	Negative test for antinuclear antibody (ANA) and rheumatoid factor

In AOSD, autoantibodies are often negative, as per the Yamaguchi criteria. Nonetheless, several autoantibody-positive AOSD patients have been reported [6,13]. After doing a retrospective analysis of 61 Chinese patients with AOSD, Zeng et al. found that seven of the 61 patients (11.5%) had positive ANA results [14]. Rheumatoid factor, anti-cyclic citrullinated peptide antibody, anti-SS-A/Ro antibody, anti-dsDNA antibody, and anti-neutrophil cytoplasmic antibodies are among the other immunological abnormalities that have been reported in individuals with AOSD [6,15,16]. There have been instances of AOSD's overlap with other rheumatic disorders, including systemic sclerosis and Sjogren's syndrome [17-19].

Our patient's diagnosis of AOSD was based on the Yamaguchi criteria, following the exclusion of other potential conditions like infection and hematologic malignancy. Although she tested positive for antinuclear antibodies (ANA), there were no specific antibodies indicative of systemic lupus erythematosus (SLE) or signs of organ involvement typically seen in SLE. The presence of a positive ANA might be linked to autoimmune thyroiditis, evidenced by elevated Anti-TPO titers. This highlights the importance of considering alternative diagnoses like AOSD, even in cases where positive Rheumatoid factor and ANA are present. It underscores the necessity for a thorough assessment to ensure accurate diagnosis and appropriate treatment.

Elevated ferritin levels are commonly seen in patients diagnosed with AOSD. A serum ferritin level that is five times above the normal range (normal range 30-400 ng/ml or above 1,000 ng/ml) is often indicative of AOSD, although this criterion has a specificity of only 41-46% [8]. Recent studies have shown that high serum ferritin levels are typically associated with disease activity and may be linked to chronic recurrent flares and reactive hemophagocytic lymphohistiocytosis. Interestingly, despite our patient having high ferritin levels, she responded well to treatment and had a positive treatment outcome.

The severity of the condition determines the course of treatment for AOSD. Inducing remission and managing current illness are two benefits of corticosteroids. NSAIDs are useful in treating mild instances, as well as in reducing fever and articular symptoms. A further course of treatment for complex and unresponsive patients is the use of biologicals like anakinra, tocilizumab, tumor necrosis factor (TNF)-inhibitors (adalimumab, etanercept, etc.) [8].

## Conclusions

In conclusion, ANA can be positive in patients of adult-onset Still's disease (AOSD). The role of classification criteria in clinical decision-making at the bedside is limited. The presence of a positive ANA in some AOSD cases underscores the importance of distinguishing between autoinflammatory (AOSD) and autoimmune diseases (systemic lupus erythematosus/rheumatoid arthritis) due to differences in pathophysiology, disease course, complications, management, and prognosis.
